# Otitis Media in Sperm-Associated Antigen 6 (*Spag6*)-Deficient Mice

**DOI:** 10.1371/journal.pone.0112879

**Published:** 2014-11-13

**Authors:** Xiaofei Li, Lei Xu, Jianfeng Li, Boqin Li, Xiaohui Bai, Jerome F. Strauss, Zhibing Zhang, Haibo Wang

**Affiliations:** 1 Otolaryngology-Head and Neck Surgery, Provincial Hospital Affiliated to Shandong University, Jinan, Shandong Province, PR China; 2 Shandong Provincial Key Laboratory of Otology, Jinan, Shandong Province, PR China; 3 Department of Obstetrics and Gynecology, Virginia Commonwealth University, Richmond, Virginia, United States of America; 4 Department of Biochemistry and Molecular Biology, Virginia Commonwealth University, Richmond, Virginia, United States of America; Zhejiang University, China

## Abstract

Mammalian SPAG6 protein is localized to the axoneme central apparatus, and it is required for normal flagella and cilia motility. Recent studies demonstrated that the protein also regulates ciliogenesis and cilia polarity in the epithelial cells of brain ventricles and trachea. Motile cilia are also present in the epithelial cells of the middle ear and Eustachian tubes, where the ciliary system participates in the movement of serous fluid and mucus in the middle ear. Cilia defects are associated with otitis media (OM), presumably due to an inability to efficiently transport fluid, mucus and particles including microorganisms. We investigated the potential role of SPAG6 in the middle ear and Eustachian tubes by studying mice with a targeted mutation in the *Spag6* gene. SPAG6 is expressed in the ciliated cells of middle ear epithelial cells. The orientation of the ciliary basal feet was random in the middle ear epithelial cells of *Spag6*-deficient mice, and there was an associated disrupted localization of the planar cell polarity (PCP) protein, FZD6. These features are associated with disordered cilia orientation, confirmed by scanning electron microscopy, which leads to uncoordinated cilia beating. The *Spag6* mutant mice were also prone to develop OM. However, there were no significant differences in bacterial populations, epithelial goblet cell density, mucin expression and Eustachian tube angle between the mutant and wild-type mice, suggesting that OM was due to accumulation of fluid and mucus secondary to the ciliary dysfunction. Our studies demonstrate a role for *Spag6* in the pathogenesis of OM in mice, possibly through its role in the regulation of cilia/basal body polarity through the PCP-dependent mechanisms in the middle ear and Eustachian tubes.

## Introduction

Otitis media (OM), inflammation of the middle ear, is the most prevalent disease in children but may occur at any age. OM can be acute or chronic and accompanied by excessive middle ear effusion, an accumulation of fluid in the middle ear [1], [2]. The etiology of OM with effusion (OME) is highly heterogeneous and thought to result from a broad spectrum of pathological mechanisms. Vulnerability to OM is attributable to bacterial infections, cilia defects and Eustachian tube dysfunction, as well as other poorly understood factors, including genetic susceptibility [3], [4].

The epithelium of the middle ear and Eustachian tube, which comprises multiciliated cells (MCCs) and non-MCCs, is closely connected with OME. MCCs project hundreds of cilia, and the directional and coordinated beating of these cilia clears contaminants from the middle ear cavity to the nasopharynx through Eustachian tube. Mucociliary clearance requires uniform cilia orientation within individual cells, among neighboring cells, and along the tissue axis [5]. Each motile cilium consists of a “9+2” axoneme and a basal body that anchors the cilium to the apical cell membrane. These motile cilia transport serous fluid and mucus of the middle ear, which plays an important role in the protection against infection [6]. As noted above, defects in cilia function cause effusion and inflammation in the middle ear, such as primary ciliary dyskinesia and Kartagener's syndrome [7], [8].

Establishment of common orientation in a sheet of cells is required for proper tissue development and function. Planar cell polarity (PCP) signaling refers to the mechanism(s) responsible for providing the cells with the information on this polarity [9]. A set of evolutionarily conserved genes, known as core PCP genes, play a vital role in the planar polarity of all tissues with PCP features. PCP proteins not only regulate cilium orientation or positioning [10], but also regulate basal body docking to the apical surface during ciliogenesis [11].

Sperm associated antigen 6 (SPAG6) is the orthologue of *Chlamydomonas PF16*. Dysfunction of *PF16* gives rise to paralyzed flagella in *Chlamydomonas* [12] It has been reported that *Spag6-*deficient mice develop hydrocephalus, and the males are sterile due to sperm motility defects [13], [14]. Recent investigation indicated that SPAG6 also regulates ciliogenesis and cilia polarity of epithelial cells in the brain ventricles and trachea [15]. Beside the upper and lower respiratory tracts, ciliated cells are also present in the middle ear cavity and the Eustachian tubes. We hypothesize that *Spag6* may play a role in maintaining normal function of middle ear and Eustachian tubes. To test this hypothesis, we examined SPAG6 expression in mouse middle ear and Eustachian tubes, and determined whether *Spag6* defects can lead to cilia and/or cilia-related structural abnormity in ciliated columnar epithelia of the middle ear and Eustachian tube. We discovered that SPAG6 is expressed in the cilia of epithelial cells in the middle ear. In the absence of SPAG6, mice developed OM, which is likely due to impaired cilia function of epithelial cells in the middle ear and Eustachian tubes.

## Materials and Methods

### Mouse husbandry and genotype

The *Spag6* knock-out mice were generated and crossed as described previously [13]. In order to obtain homozygous (*Spag6^−/−^*) and wild-type (*Spag6^+/+^*) mice, male and female heterozygous mice were crossed, and offspring of these mice were genotyped by PCR as described previously [13]. Use of animals for these experiments was approved by the Ethics Committee of the Provincial Hospital Affiliated with Shandong University.

### Tissue preparation for histological analysis

For harvesting of bullae tissues, the animals were deeply anesthetized with 10% chloral hydrate (0.004 l/kg). After the mice reached a surgical level of anesthesia, bullae (including both middle and inner ear) were isolated from ears of control (n = 4) and *Spag6* mutant mice (n = 4) at each time point (postnatal days 9, 12, 18, 30, 180). After dissection, bullae were fixed in 4% paraformaldehyde (PFA) for 24 hours and then decalcified in 10% EDTA for 3 days and dehydrated with the different concentration ethanol. After dehydration fully, the tissues were embedded in paraffin for sectioning. Sections were counterstained in hematoxylin/eosin (H&E) and Mucicarmine staining Kit (BA-4086A, Baso, China).

### Transmission Electron Microscopy (TEM)

Mice were sacrificed and cardiac perfused by 2% glutaraldehyde. Fixed middle ear mucous epithelium were separated and dehydrated in ascending concentrations of ethanol, embedded in Epon resin and sectioned. Ultrathin sections were used to determine the right position. Ultrathin sections were stained with lead citrate and uranyl acetate, and examined using a JEM-1200 EX electron microscope (FEI, Hillsboro, USA).

### Scanning electron microscopy (SEM)

The bullae from *Spag6^−/−^* and wild-type mice were dissected and then immersed in 2.5% glutaraldehyde in 0.1 M phosphate buffered saline (PBS, pH = 7.2) overnight at 4°C. After exposure of the middle ear cavities, a post-fixation in 1% OsO_4_ in 0.1 M PBS (pH = 7.2, 1 hour) was performed. Samples were washed in 0.1 M PBS (pH = 7.2), dehydrated and critical point dried, coated with approximately 10 nm of platinum, and examined under a Hitachi S-900 scanning electron microscope (Hitachi Ltd, Tokyo, Japan) at 15 kV.

### Bacterial identification and colony counting

The middle ears were isolated under sterile conditions and washed with sterile PBS. 100 µl of the PBS lavage were inoculated onto a chocolate agar (JINAN BABIO BIOTECHNOLOGY CO. LTD, China), and spread the plate with a spreader. After 20 minutes' standing, the plates were reversed and incubated at 37°C with 5% CO_2_ for 24 hours. Bacterial colonies were counted and then collected. The bacterial DNA was extracted using a bacterial DNA kit (D3350, Omega Bio-tek, Inc, Norcross, USA) for further identification by PCR [16]. *Streptococcus pneumonia* (American Type Culture Collection (ATCC), Manassas, VA, USA), *Haemophilus influenza* (ATCC49247, Microbiologics, Inc. Minnesota, USA), and *Moraxella catarrhalis* (ATCC25240, Microbiologics, Inc. Minnesota, USA) were used as positive controls.

### Immunohistochemistry

After being dewaxing and hydration, the samples were washed in 0.01 M PBS and blocked in 10% goat serum albumin and 0.1% TritonX-100 for 1 hour at room temperature, then incubated in rabbit anti-SPAG6 antibody at 4°C overnight. After being washed fully in PBS, the tissues were incubated with secondary antibodies (dylight 649 goat anti-rabbit, EarthOx Life Science, Millbrae, USA, 1∶100) in block solution at the room temperature for 1 hour, the counterstained with 4′,6-diamidino-2-phenylindole (DAPI) (Molecular Probes, Inc, Eugene, USA) for 15 minutes and phalloidin for 20 minutes. The samples were observed under a laser scanning confocal microscope (DMI 4000B, Leica Microsystems Ltd, Beijing CN-100044, China).

### Middle ear epithelium stretched preparation

Bullae were isolated from ears of control (n = 4) and *Spag6* mutant mice (n = 4) aged 25 days and fixed in 4% PFA for 24 hours. The middle ear epithelium were peeled off the bone carefully under the dissecting microscope (LEICAL2, Leica Microsystems Ltd, Beijing CN-100044, China). The samples were washed in 0.01 M PBS and subjected to the same steps as described for immunohistochemistry methods. The primary and secondary antibody were goat anti-mouse FZD6 antibody (AF1526, R&D Systems, Inc., Minneapolis, MN 55413 USA,1∶40) and Dylight 405 rabbit anti-goat IgG antibody (EarthOx, LLC, San Francisco, CA, USA, 1∶100), respectively.

### Skull preparation and craniofacial measurement

The skulls of the wild-type (n = 8) and mutant (n = 8) mice were dissected and immersed in 1% potassium hydroxide to clear the soft tissues and then stained with alizarin red [17]. The angle between the midline of the skull base and the bony part of the Eustachian tube was measured for *Spag6* mutants and wild-types.

### Quantitative real-time reverse transcription-polymerase chain reaction (qPCR)

The mucosa of the middle ears were collected from the mutant and wild-type mice and total RNAs were isolated from each tissue sample using Trizol reagent according to the manufacturer's instructions (Invitrogen, Carlsbad, CA, USA). The cDNA was synthesized from each RNA sample using cDNA Synthesis Kit (Ta Kara Bio, Siga, Japan). QPCR was performed using SYBR Green PCR kits and an Eppendorf AG 22331 Hamburg machine (Germany). Primers used for real-time PCR are reported previously [16].

### Statistical analyses

Values are reported as means ± standard error of mean (SEM). Significant differences were determined by the Student t test. Values of P<0.05 were considered to indicate statistical significance. Eustachian tube angles were measured using Image J software (National Institutes of Health, USA).

## Results

### SPAG6 is expressed in the cilliated epithelium of the middle ear

**Figure 1:**
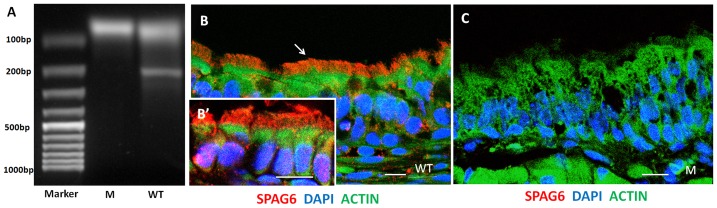
SPAG6 is expressed in the cilliated epithelium of the middle ear. RT-PCR and immunofluorescence were conducted to detect the expression of SPAG6 respectively. (A) RT-PCR showed that the 190-bp amplicon representing the *Spag6* mRNA was detectable in the middle ears of the wild-type mice. No amplicon was detectable in the *Spag6*-deficient mice. (B) The immunofluorescence staining showed that SPAG6 (red) is located in the cilia of the ciliated columnar epithelium. (C) No staining could be detected in *Spag6*-deficient mice. DAPI (blue) and phalloidin (green) staining were used to outline the cell nucleus and cytoskeleton respectively. M, mutant; WT, wild-type. Bars: 10 µm in B–C; 7.5 µm in B'.

RT-PCR amplified a 190-bp amplicon representing the *Spag6* mRNA from the middle ear tissues of the wild-type mice. No amplicon was detectable in the *Spag6*-deficient mice (Figure 1A). Immunofluorescence staining demonstrated that SPAG6 was located in the cilia of the ciliated columnar epithelium (Figure 1B). No staining could be detected in *Spag6*-deficient mice (Figure 1C).

### Otitis media in *Spag6*-deficient mice

**Figure 2:**
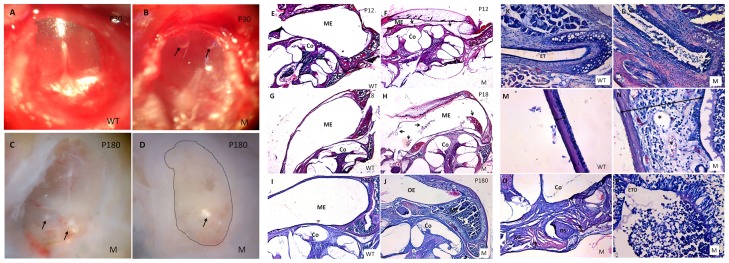
Otitis media is observed in *Spag6*-deficient mice. (A–B) Effusion and two bubbles (arrows) could be seen in the mutant mice. (C–D) Some crystals (arrows) were found in a mutant mouse aged 6 months. Fibrous tissue fills the whole middle ear cavity (dotted box). (E–F) At P12 (n = 4, each genotype), the mutant mice did not show otitis media with effusion (OME), some mesenchymal cells (arrows in F) could be observed in the middle ear cavity (ME). (G–H) At P18 (n = 4, each genotype), inflammatory cells began to show in the middle ears of the mutant mice (arrows in H). (I–P) At P180 (n = 1, each genotype), inflammatory cells and effusion were full of the ME(J) and Eustachian tube (ET) (L), Eustachian tube opening (ETO) was blocked by the inflammatory effusions (P). The middle ear epithelium also became thickened in the mutant mice (N, two-ended arrow). Otosteon (OS) was fixed by the surrounding cholesterol crystals (O). Co, cochlea; OE: outer ear; M, mutant; WT, wild-type. (5× magnification, E–J; 20× magnification, K–L; 40× magnification, M–P).

To explore the potential role for *Spag6* in the middle ear of mice, *Spag6^−/−^* mice and wild-type littermates were studied at five time points from postnatal days 12 (P12) to 6 months of age (four mutant and four wild-type at each time point), and their ears were examined by otoscope and histological sections with H&E staining. Before 12 days (Figure 2E, F), although mesenchymal tissue still existed, cavitation of the middle ear was larger and appeared to proceed earlier in wild-type mice than in mutants. By 18 days (Figure 2G, H), all the mutant mice had bilateral effusion in the middle ear cavity or/and Eustachian tube, thickened tympanic mucosa with inflammatory cells, indicating that the mutant mice have manifestations of OM. The trend continued in 30-day-old mutant mice. At 30 days (Figure 2A, B), effusions had spread throughout the middle ear cavities of mutant mice as OM progressed. Wild-type mice at 30 days showed no OM pathology. Almost all mutant mice die from hydrocephalus by six months of age, but in one mutant mouse surviving to 6 months, the previously noted middle ear pathology was accompanied by capillary hyperplasia, cholesterol crystals, fixation of otosteon and fibrosis (Figure 2C, D, I–P), indicating that tympanosclerosis is secondary to OM.

### Analyses of cilia in the epithelial cells of the middle ear and Eustachian tube

**Figure 3:**
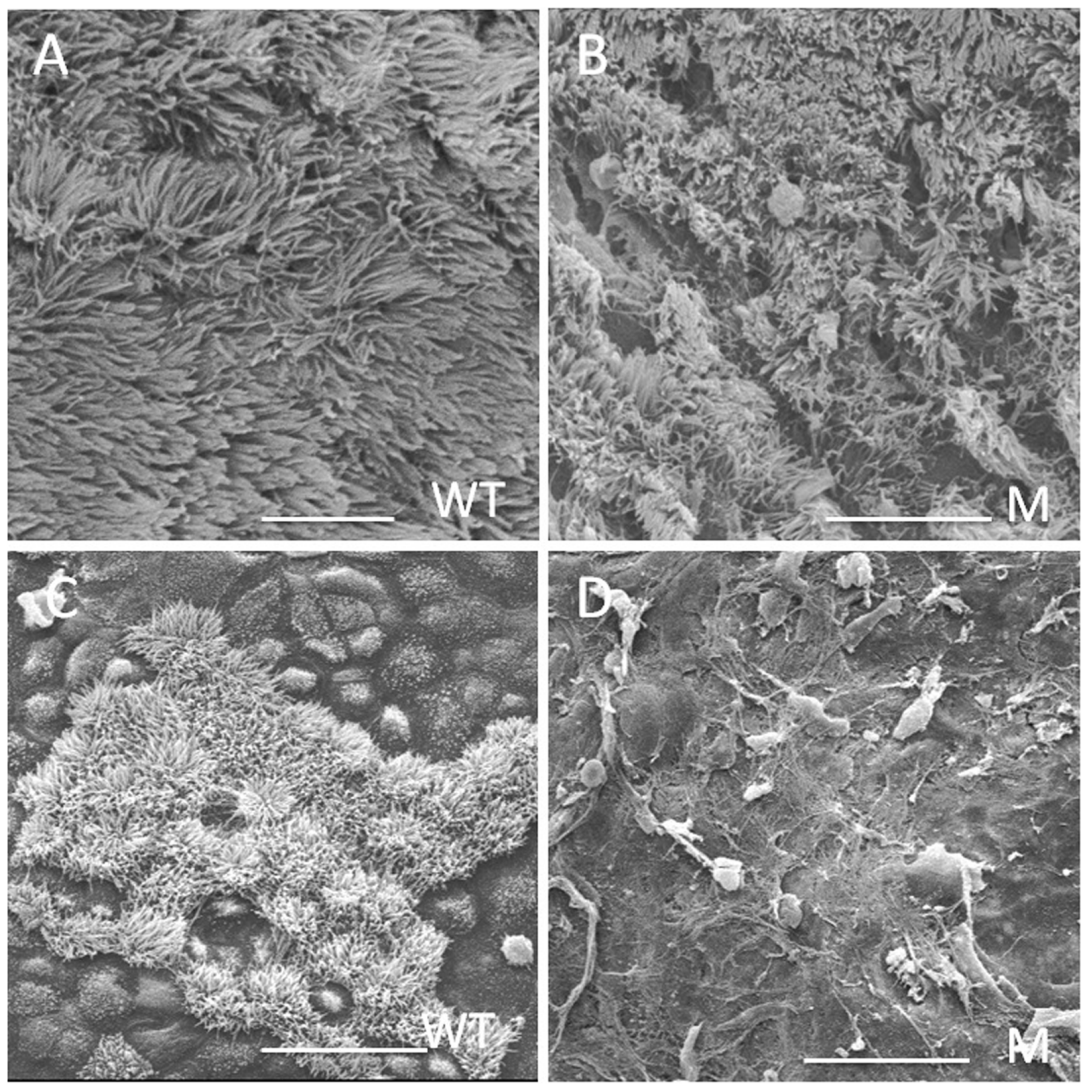
Analyses of cilia in the epithelial cells of the middle ear and Eustachian tubes. (A) Normal-looking cilia in the middle ear epithelial cells of 25-day old wild-type mice, the cilia were well organized. (B) Cilia in the middle ear epithelial cells of 25-day old *Spag6-*deficient mice, notice that the cilia were largely disordered. Cilia were present in the promontorium tympani of 25-day old wild-type mice (C), but were prematurely lost in the *Spag6-*deficient mice (D). WT, wild-type; M, mutant; Bars: A, 10 µm; B, 15 µm; C and D, 25 µm.

SEM result showed that cilia of the wild-type mice (at P25) were well-organized in the middle ear epithelial cells (Figure 3A). However, there was significant reduction in cilia density of the *Spag6-*deficient mice (at P25), and the cilia were largely arrayed in a disordered fashion (Figure 3B). Cilia were present in the promontorium tympani of the wild-type mice (Figure 3C), but, they prematurely disappeared in the *Spag6-*deficient mice (Figure 3D).

### The orientations of basal beet and two central microtubules were disrupted in middle ear epithelial cells in *Spag6*-deficient mice

**Figure 4:**
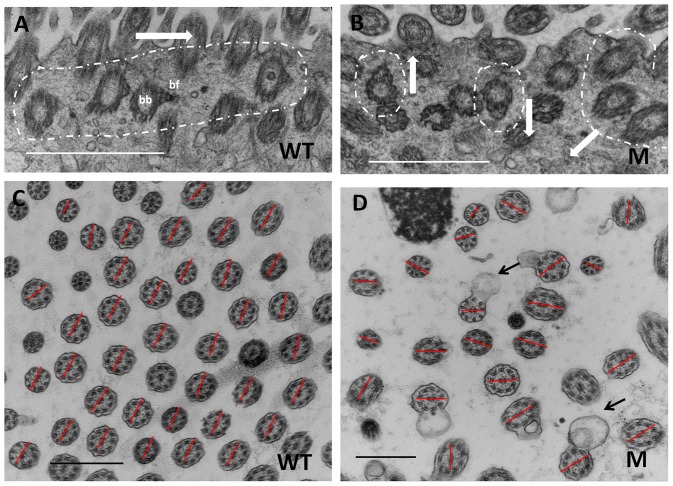
Orientations of basal beet and two central microtubule were disrupted in the middle ear epithelium of *Spag6-*deficient mice. (A, B) The basal feet of middle ear ciliated epithelium in 25-day old wild-type mice and *Spag6^−/−^* mice. (C, D) The orientations of the two central microtubules of the cilia in 25-day old wild-type mice and *Spag6^−/−^* mice. (A) In the wild-type, the basal feet point in the same orientation (arrow and dotted circle in A); (B) while in the mutant mice, the orientation of basal feet was disrupted and appeared to be random (arrows and dotted circles in B). (C) In wild-type mice, the orientation of the two central microtubules of the cilia was consistent, as shown by the same orientation of lines connecting the two microtubules (red lines in C). (D) However, in *Spag6*-deficient mice, the orientation of the two central microtubules was random; the lines connecting central microtubules pointed to different directions (red lines in D). Moreover, abnormal ciliary membranes (black arrows in D) also could be seen. bb, basal body; bf: basal foot; WT, wild-type; M, mutant; Bars in A and B, 1 µm; Bars in C and D, 500 nm.

Basal feet orientations indicate cilia orientations and tissue level polarity. Our observations revealed that in the 25 day old wild-type mice, orientation of basal feet of middle ear epithelial cells was similar, with the basal feet all pointing in the same orientation (Figure 4A). However, in the mutant mice (at P25), the orientation of basal feet appeared to be random (Figure 4B). Consistent with the basal feet results, in wild-type mice, the orientation of the two central microtubules of all the cilia was uniform, as shown by the same orientation of lines connecting the two microtubules in all the axonemes (Figure 4C). However, in *Spag6*-deficient mice, the orientation of the two central microtubules was evidently random, with the lines connecting central microtubules pointing in different directions in some axonemes (Figure 4D).

### FZD6 protein does not have a polarized distribution in mutant tympanic ciliary epithelium

**Figure 5:**
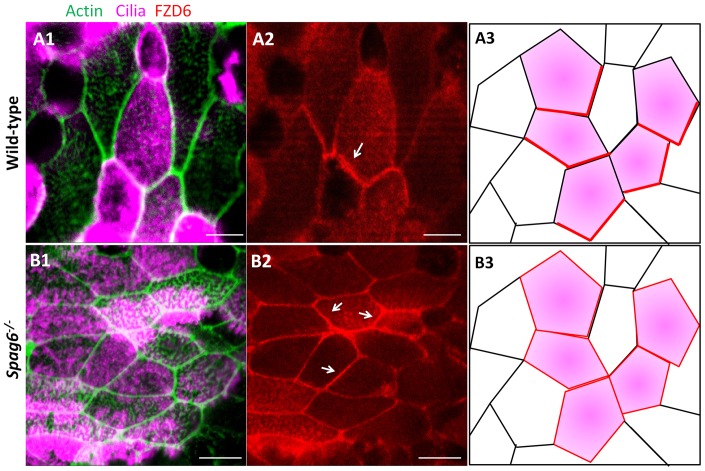
FZD6 protein polarity is lost in mutant tympanic ciliary epithelium. (A–B) The distribution of the core PCP protein, FZD6, was examined by immunofluorescence staining. Ciliated epithelial cells were marked with anti-acetylated-α-tubulin antibody (rose). (A1, A2) In the wild-type mice (at P25), FZD6 protein localized asymmetrically to the cell cortex at the level of the apical junctions (arrow in A2). (B1, B2) However, in the mutant mice (at P25), FZD6 protein lost its polarized distribution and was located on the entire membrane at the apical level (arrows in B2).(A3, B3) Schematic diagram of FZD6 protein (red line) distribution in wild-type and *Spag6^−/−^* mice. The color codes are the same as that in panel A1 and B1. Bars: A, 5 µm; B, 7.5 µm.

Mis-oriented basal feet imply a PCP defect. Therefore, localization of FZD6, a core PCP protein, was examined by immunofluorescence staining. In the 25 day-old wild-type mice, FZD6 protein was localized asymmetrically to the cell cortex at the level of the apical junctions (Figure 5A). However, in the mutant mice (at P25), FZD6 protein lost its polarized distribution and appeared to be located throughout the entire apical membrane (Figure 5B).

### Moraxella catarrhalis is detected both in the mutant and wild-type mice

**Figure 6:**
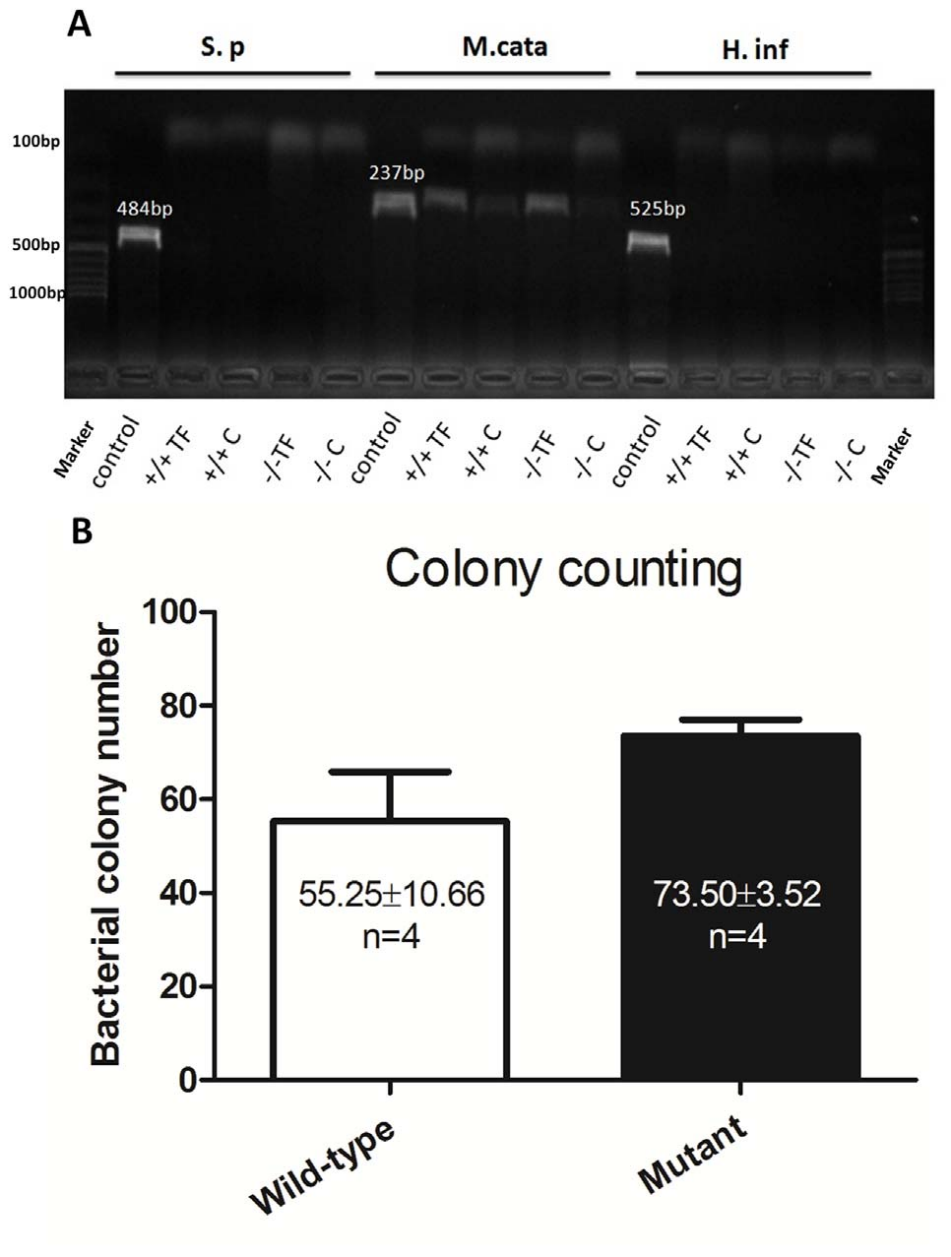
Moraxella catarrhalis is detected both in the mutant and wild-type mice. (A–B) PCR results and bacterial colony count results. (A) Specific primers for *Streptococcus pneumonia (S.p), Haemophilus influenza (H.inf)*, and *Moraxella catarrhalis (M.cata)* were used to detect the bacteria in the middle ear. Bacteria obtained from ATCC were used as the reference. *Moraxella catarrhalis* was detected (237 bp, n = 4 for each group) in the tympanic washing fluid from both of the 25 day old mutant mice and control mice. *Moraxella catarrhalis* was also detected in cultures in both wild-type mice and mutant mice, which was consistent with PCR results. The colony count results showed that there was no significant difference in quantity of bacterium between the two groups (P>0.05). *Streptococcus pneumonia* (484 bp) and *Haemophilus influenza* (525 bp) were undetectable. +/+, the wild-type mice; −/−, the mutant mice; TF, tympanum fluid; C, culture.

To assess whether the OM in *Spag6^−/−^* mice is caused by bacteria infection, PCR and bacterial culture were conducted to identify pathogens in the middle ear. Three bacteria species (*Streptococcus pneumoniae, Haemophilus influenzae, and Moraxella catarrhalis*), which are the most common bacteria seen in OM, were tested. *Moraxella catarrhalis* were detected by PCR (n = 4 for each group) in the tympanic washing fluid from both mutant mice (at P25) and wild-type mice (at P25) (Figure 6A). *Moraxella catarrhalis* was also detected in cultures, consistent with PCR results. Colony counts of cultures were performed to compare the difference between the wild-type and mutant mice. There was no significant difference in the colony count between the two groups (P>0.05) (Figure 6B). *Streptococcus pneumonia* and *Haemophilus influenza* were not detected. However, these observations do not preclude infection by other microorganisms not specifically targeted for analysis as a cause of OM.

### No obvious changes in epithelial goblet cell density and mucin expression in middle ears of mutant mice

**Figure 7:**
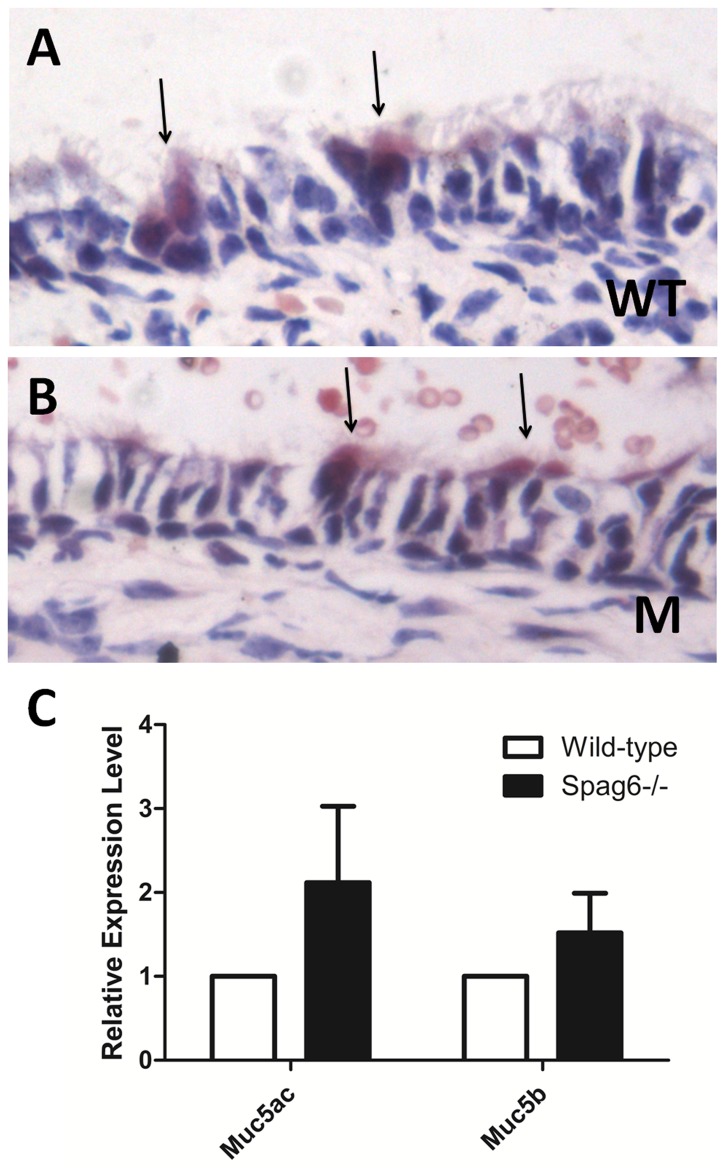
No obvious changes in epithelial goblet cell density and mucin expression in middle ears of mutant mice. (A–B) Mucicarmine staining shows rose-stained goblet cells (arrows) in the wild-type (A) and mutants (B). There was no significant difference in goblet cell density between the wild-type and the mutants. (C) Analysis of expression level of *Mucin5ac* and *Mucin5b* by real-time PCR. There was no statistical significance in expression between the wild-type and the mutant mice (n = 8, each genotype, P>0.05). WT, wild-type; M: mutant. Muc5ac, Mucin5ac; Muc5b, Mucin5b.

It has been reported that expression of the mucin gene family is affected in several mouse models of OM [18], [19]. Mucins, secreted by middle ear epithelial goblet cells, are heavily glycosylated proteins that are considered primarily responsible for the gel-like characteristics of mucoid middle ear fluids. Mucin5ac and Mucin5b are the key molecules that determine the properties of airway mucus gel [20], which have been reported to be involved in the development of OM and inflammation. Epithelial goblet cells and mRNA expression of *Mucin5ac* and *Mucin5b* were examined by mucicarmine staining and real-time PCR, respectively. The number of goblet cells in the middle ear cavity and Eustachian tubes appeared to be similar in the mutant and wild-type mice. There were also no significant differences in the expression of *Mucin5ac* and *Mucin5b* in the middle ear (Figure 7).

### Eustachian tube angle is normal in *Spag6-*deficient mice

**Figure 8:**
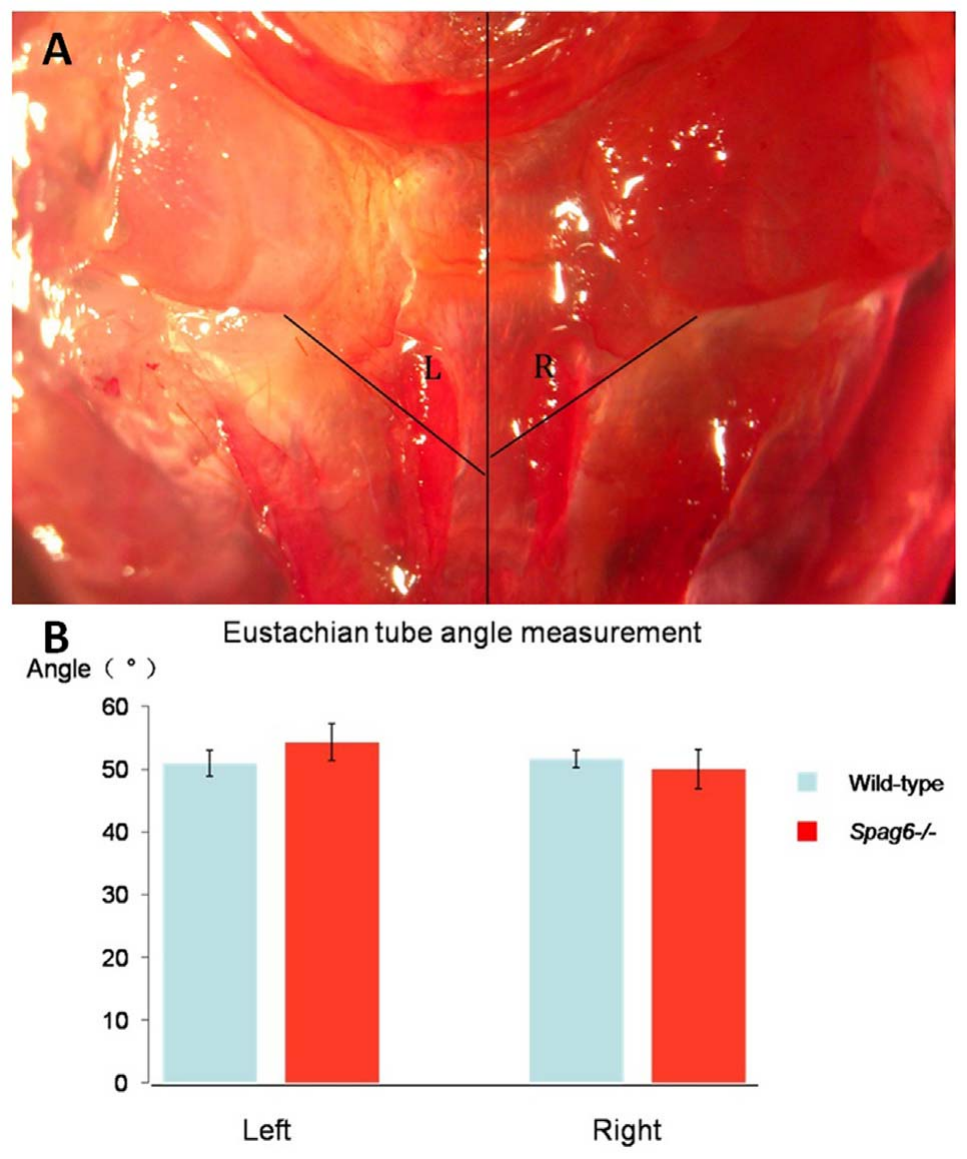
Normal Eustachian tube angle in *Spag6-*deficient mice. (A) The panel showed a direct view of a dissected skull with the lines of reference for the measured angles indicated. (B) The panel showed a graphic comparison of the mean values for angles L and R between mutant (n = 8) and wild-type (n = 8) mice. There is no significant difference in Eustachian tube angles between the wild-type and the mutant mice (Student t test, P>0.05). Error bars indicate the SEM.

Eustachian tube abnormalities are often related to the increased OM incidence. Thus, Eustachian tube angles were measured between *Spag6-*deficient mice and their wild-type littermates to determine whether Eustachian tube angle contributes to OM in mutant mice. There was no significant difference between the mutant mice and the wild-type mice, indicating normal Eustachian tube angle in the mutants (Figure 8).

## Discussion

SPAG6 was originally reported to play a role in regulating cilia motility [13], [14]. Recent studies demonstrated that SPAG6 also plays a role in ciliogenesis and polarity of epithelial cells in the brain vectricles and trachea [15]. Epithelial cells in the middle ear and Eustachian tube also have motile cilia [21]. However, it was known whether SPAG6 has a role in themiddle ear, which has a ciliated epithelium. In the current study, we discovered for the first time, that *Spag6* is expressed in the epithelial cilia of the mouse middle ear, and that a targeted *Spag6* mutation results in middle ear pathology (OM), that can be attributed to ciliary dysfunction.

To examine the relationship between middle ear cavity formation and OM progression, the time-course of OM pathology progression was studied. This revealed that 18-day-old mutant mice have middle ear effusions and inflammation (a primary sign of OM), while no manifestations of OM are observed in the wild-type littermates of a similar age, suggesting that *Spag6* mutation exerts influences as early as middle ear cavity just formtion. By 6 months, mutant mice showed capillary hyperplasia, cholesterol crystals, fixation of otosteon and fibrosis, which is a sign of tympanosclerosis, usually secondary to OM. These phenotypes are consistent with findings in humans, raising the possibility that mice with *Spag6* mutations could be useful models for the study of genetic factors contributing to OM [22], [23].

Impaired mucocilliary function is a common factor that contributes the pathogenesis of OM [3], [24], [25]. Mucociliary clearance requires uniform cilium orientation within individual cells, and along the tissue axis [5]. Ciliary basal feet, basal body appendages, point proximally in the direction of the ciliary active stroke [26]. The cilia axoneme central microtubules play important roles in the stability of axoneme structure and coordination of ciliary beating. In our study, although the “9+2” axonemes of cilia in middle ear and Eustachian tubes were conserved in most cases, the orientation of central microtubules were not uniform, as was the orientation of the ciliary basal feet, indicating that as in brain and lungs, *Spag6* mutation affects the polarity of the middle ear epithelium with the gross structure of the “9+2” axonemes being conserved [14], [15]. However, we cannot rule out the possibility of subtle ultrastructural abnormalities in the axonemes that may go undetected at the level of resolution of our TEM studies. SEM studies demonstrated that cilia number was also reduced and confirmed the disordered cilia orientation in mutant mice. These findings predict abnormal ciliary beating in the middle ear and Eustachian tubes of mutant mice. Therefore, we propose that *Spag6* inactivation leads to abnormal ciliary motility and diminished fluid and mucous transport, which upsets the balance between fluid and mucin secretion and clearing, resulting in middle ear effusions and OM.

In addition to cilia dysfunction, OM can be caused by bacteria, excessive mucus secretion [27], and Eustachian tube abnormalities [28], [29]. Although *Moraxella catarrhalis* was detected at a similar level in both wild-type and mutant mice, only the mutant mice had manifestations of OM. However, our studies cannot exclude the possibility that other microorganisms contributed to OM in the mutant mice. The mutant mice did not show evidence of greater mucous production compared to wild-type mice (similar density of goblet cells and similar level of mucin gene expression). Furthermore, Eustachian tube angle measurement did not reveal any abnormality in the mutant mice. Thus, it is unlikely that OM in *Spag6*-deficient mice is caused by *Moraxella catarrhalis*, mucus and Eustachian tube angle defects, consistent with the notion that impaired cilia function might be responsible for spontaneous OM in the mutant mice.

FZD6 lost its polarized distribution in the middle ear epithelium of *Spag6*-deficient mice, indicating that *Spag6* inactivation altered the location of FZD6 protein in the middle ear, and that planar polarity of the middle ear were affected. This is consistent with the report that FZD6 knockout mouse have defects in the planar polarity of the inner ear and neural tube closure [30]. Our observation raises the possibility that *Spsg6* may have a role in regulating PCP in the middle ear. However, it is also possible that the disordered cilia arrays in the mutant mice are secondary to ciliary beat defects and the resultant abnormal fluid flow caused by the loss of SPAG6 from the axoneme central apparatus.

Studies have shown that the polarized array of microtubules contributes to proper distribution of the PCP regulators. Disruption of microtubules perturbs the localization of Frizzled in Drosophila wing epithelium. Therefore, polarized transport of core PCP proteins depends on the planar microtubule arrays [31]. Furthermore, planar polarized microtubules not only participate in orienting and spacing basal bodies within a meshwork in each cell, but also transmit directional information from PCP proteins to basal bodies, orienting them in the direction of PCP signaling [5]. Our previous work found that SPAG6 is a microtubule binding protein [32], and *Spag6* knockout leads to instability of microtubules in the axoneme [13]. Based on the important roles of microtubules in planar polarity, and the interactions between SPAG6 and microtubules, we hypothesize that defects in the planar polarity of middle ear may involve unstable microtubules system by *Spag6* inactivation. In line with this hypothesis, we showed abnormal FZD6 protein distribution in the mutant mice and disordered orientations of basal bodies and axoneme central apparatus.

In summary, our findings demonstrate an association between *Spag6* mutation and the pathogenesis of OM in mice. OM appears to develop as a result of ciliary dysfunction leading to effusions and progressive middle ear inflammation. The structural abnormalities in cilia orientation may be a consequence of *Spag6*′s contribution to the regulation of PCP in the middle ear and Eustachian tubes.
